# Exercise in Aging: Be Balanced

**DOI:** 10.14336/AD.2021.0107

**Published:** 2021-08-01

**Authors:** Joanna Gronek, Michał Boraczyński, Piotr Gronek, Dariusz Wieliński, Jacek Tarnas, Sławomir Marszałek, Yi-Yuan Tang

**Affiliations:** ^1^Department of Dance, Poznań University of Physical Education, Poland.; ^2^Faculty of Health Sciences, Collegium Medicum, University of Warmia and Mazury, Poland.; ^3^Department of Anthropology and Biometry, Poznań University of Physical Education, Poland.; ^4^Department of Physical Education and Lifelong Sports, Poznań University of Physical Education, Poland.; ^5^Department of Physiotherapy, Poznań University of Physical Education, Poland.; ^6^Department of Physiotherapy, Poznań University of Medical Sciences, Poland.; ^7^Department of Psychological Sciences, Texas Tech University, USA.

**Keywords:** older people, healthy aging, physical activity, physical activity pattern, mindfulness and exercise

## Abstract

The beneficial effects of exercise are recognized for preventing physical and cognitive decline during the aging process. However, there is still a gap concerning recommended intensity, volume, frequency and mode of exercise especially for older people. The aim of this study was to investigate an appropriate type of physical activity (PA) model for healthy aging. A commentary of the influence of PA and exercise on healthy aging through an online search of the databases Web of Science, PubMed and Google Scholar. Two living groups can be considered as potential references: modern hunter-gatherer small-scale population and master athletes. Greater physical activity is proposed for healthy aging than that recommended by WHO. Additionally, mindfulness meditation techniques during exercise are recommended especially for persons practicing long-duration exercises. Complex and compound exercise and workouts should include challenging exercises adjusted and balanced to provide clients, especially older people, with noticeable changes and progress.

By the year 2030 elderly adults over 65 years of age is estimated to comprise 20% of the population [1], and by 2050 the number of people 60 years old and over worldwide to be 21.1% or 2 billion [2]. Because of the increasing aging population in many countries throughout the world, much evidence shows that pharmacological treatment, in some instances, is indispensable during the aging process. However, a non-pharmacological approach can be just as effective if not more given it does not disrupt the normal bodily function of interest and be used in the prevention or management of the disease at hand. Exercise and general physical activity (PA), a prominent non-pharmacological treatment is a promising, low-cost, low-risk and widely available, which is recognized for its reduction impact in cancer, cardiovascular diseases, vascular aging, cardiorespiratory fitness, psycho-motor skills, mood and health-related quality of life (HRQL) [3,4,5,6]. Moreover, the beneficial effects of habitual exercise and PA on brain structure (cortical and sub-cortical regions), brain function (neural plasticity) [7,8] and consequently on cognition and psychological well-being have been promising elements of in preventing brain deterioration and cognitive decline during the aging process [9,10,11,12,13]. Finally, PA levels are shown to ensure subjective well-being among older adults [14] which is associated with healthy aging and longevity [15].

## Types of exercise

According to Baar, exercise can broadly be grouped into subclasses: aerobic/endurance and strength/resistance [16]. An additional subgroup that should be noted is patterned movements. While resistance and endurance exercise significantly influence muscle phenotype, patterned movement exercises are mainly a motor program in the central nervous system and result in relatively small or indistinguishable biochemical changes in myocytes [17]. Mechanisms underlying muscle adaptation to exercise have been better recognized as a model to explain the concurrent training effect [16]. The main exercise characteristics are intensity and volume. Their reciprocal relations in the decrease of one variable and simultaneous increase of another variable result in a different adaptation within the myocytes.

The results of resistance and high-resistance training in young, healthy people relate to RNA, protein content [18], fast-twitch fiber’s cross-sectional area [19,20] and the capacity to generate force [21]. Complex interventional programs including resistance training are especially important to reverse frailty and sarcopenia and consequently decrease physical disability and early cognitive dysfunction (cognitive frailty) in older persons [22, 23]. This type of exercise focuses on the sarcopenic aspects of frailty, because about 70% of frailty is due to sarcopenia [24] which is defined as low muscle function or strength in the presence of low muscle mass [25,26]. In contrast, increasing the duration and simultaneously decreasing the intensity of exercise results in different adaptations within the myocytes, including decreased glycolytic enzymes [27], increased oxidative enzymes and slow contractile and regulatory proteins [28,29], increased mitochondrial mass and capillarization [30, 31], and a decrease in the fast-fiber area [32].

All exercise stimulates brain blood flow, but this process depends on the mode (the type of activity) and intensity. During steady-state walking, cycling, running, swimming, etc. total brain blood flow increases in parallel with increasing cardiac output and VO2 consumption, although average arterial pressure remains constant [33]. Neural adaptation starts at the beginning of the exercise and gradually covers the increase in regional brain blood flow and autonomic nervous system changes. The increased brain blood flow at the beginning of exercise is due to both increased cardiac output as well as to changes in brain metabolism to supply increased neural activation. However, the decisive and most important variable is the intensity of the exercise, since carotid artery blood flow at a higher intensity continues to rise [34], which is thought to be due to the necessity to maintain thermoregulation during higher-intensity exercise [35]. Therefore, in theory in comparison to vigorous-intensity training, moderate-intensity training may be enough to result in acute augmentation of blood flow to the brain, thus achieving the equivalent myocyte adaptation that is desired.

## WHO and CSEP recommendations for physical activity

Sedentary behavior (SED) is characterized by an energy expenditure less (or equal) than 1.5 metabolic equivalents (MET) [36] while physical activity (PA) is often classified depends on exercise intensity. Vigorous-intensity PA refers to exercise that is performed at 6.0 or more times the intensity of rest for adults (>6 MET’s, where 1 MET equals to the oxygen cost of sitting quietly, an equivalent of 3.5 mL/kg/min or 1 kcal/kg/hour) while moderate-intensity PA refers to exercise that is performed at 3.0-5.9 times the intensity of rest.

The World Health Organization (WHO) considers both intensity for PA and also the frequency of weekly PA adjusted to age [37]. Therefore, adults aged 18-64 yrs and for adults > 65 yrs are recommended a minimum seventy-five minutes of vigorous-intensity aerobic PA or at least 150 min of moderate-intensity aerobic activity per week, respectively. These recommendations are important to be considered since sedentary time and total moderate-to-vigorous (MVPA) activities are associated to health risk in adults [38]. An equivalent combination of MVPA can be suggested, such as 2.5 hours of lawn-mowing t (21 min/day) or 75 min. vigorous biking (11 min/day) fulfills weekly PA energy expenditure recommended by WHO for human health as well.

These examples of recommendations seem low and somewhat conservative, and probably were dictated by the principle *primum non-nocere*. Unfortunately, approximately 30%-60% of adults aged ≥60 years across the WHO regions fail to meet the recommended PA levels [39]. Moreover, although reaching current recommended PA levels are sufficient for partially reducing risk factors for cardiovascular disease (CVD), they do not eliminate the additional risk that overweight/obesity poses [40]. In contrast to the WHO recommendations, some international PA guidelines recommend the incorporation of moderate-intensity PA on all days of the week or add other types of exercise [41]. However, due to the risk of injury with aging and problems associated with adherence, more vigorous forms of PA are recommended for experienced older adults [5]. In addition to those by WHO, the Canadian Society for Exercise Physiology (CSEP) recommends the addition of muscle and bone strengthening activities using major muscle groups at least two days per week [42].

The gap seems especially crucial in context since higher cardiorespiratory fitness is strongly associated with lower rates of age-related decline in gray matter, particularly in the prefrontal, superior parietal, and temporal cortices of cognitively healthy older adults [43,44].

Thus, the starting point in assuming that adequate PA is correlated with the cognitive decline in aging. However, a few interesting questions arise from WHO recommendations. Firstly, concerns to whom should individual recommendations be dedicated to, and should we consider sedentary humans as the “norm”? [44]. Secondly, what is the ideal model for human PA, especially in the context of healthy aging, should there be a real pattern/model? The argument arises that it is unrealistic to generalize PA by the model since each client requires a personalized approach where the aim is searching for specific exercises that will allow each client to reach the balance in their mind and body.

## What model of PA should be a real pattern?

It is widely accepted that the existing human genome was selected in the circumstances of restricted energy intake and increased demand for total energy expenditure (TEE) adjusted for hunter-gatherers Stone Age living in a rough foraging environment. That genetic constitution has stayed relatively unchanged, but nowadays TEE has changed dramatically, including motor activity, body composition with fatter tissue and less skeletal muscle tissue, and energy intake. The change in human TEE/kg/day is estimated to about 65% that of humans from the late Paleolithic Stone Age. Additionally, TEE adjusted per unit body mass of PA for contemporary Westerns is about 38% that of their ancestors [45].

Two living groups, modern hunter-gatherer small-scale populations and master athletes, seem to be considered as potential references to determine the recommended model for human PA, in the context of healthy aging. The level of hunter-gatherers TEE/kg/day is comparable only to physical fitness enthusiasts exercising the equivalent of running 7.5 mph, 60 min/day. Additionally, hunter-gatherer populations are referenced for their excellent metabolic and cardiovascular health, making them models in public health.

The Hadza hunter-gatherer small-scale population of around 1000 people from a savanna-woodland habitat near Lake Eyasi in northern Tanzania is characterized by moderate and vigorous PA levels, exceeding 100 min×d^-1^ [46]. Additionally, this population has an extremely low obesity prevalence (<5%) and a modest mean body-fat content (women: 24-28%, men: 9-18%). Metabolic CVD is not common, but longevity is an analog to that of industrialized populations. However, the Hadza people, significantly differ not only in terms of PA, but also nutrition, gut microbiota, exposure to pathogens, activity budgets and modes of subsistence. Moreover, findings from Western populations do not uniformly match findings from non-Western populations in many other types of investigation [47]. Thus, some abstinence and awareness are necessary due to certain limitations in accepting this group as a potential reference, even if it arouses highest curiosity. Master athletes with or without sports history exercise four to six days/wk can be the second model group for PA of healthy aging. This group includes athletes over 35-40 years old and are usually referred to as “veteran”, “master”, or “seniors”. This group exercises over the set point, as suggested by Lazarus and Harridge [48], a given threshold of PA that is needed to age optimally and to maximize the “healthspan”. Exercising at levels of a certain intensity, volume, frequency below the set point will result in aging being contaminated by the pathological and unpredictable effects of inactivity. Exercise over this threshold stimulates maximized athletic performance but is unlikely to have any further beneficial effects on health [48].

From a gerontological perspective, numerous studies included in a review by Lemez and Baker [49] reported a longer lifespan for elite athletes compared to age- and sex-matched controls from the general population and other athletes. The previously observed relationship between participation in elite sport and longevity [50, 51] can enhance our notion of the benefits of PA at the highest levels of competition in unique athletic cohorts. However, there is still controversy about the recommended intensity, volume, duration and frequency of PA for the elderly. Professional master athletes apply training loads at the highest values, while amateur athletes are recommended by some specialists much lower training loads at light and moderate intensity levels. Prof. Tanaka suggested slow jogging almost at a speed of fast walking to allow comfortable talking, similar to “tiptoe” running [52]. Furthermore, running is recommended for older people to reduce all-cause mortality, even if they have advanced arthritic changes. However, the distance and intensity should be adjusted to the individual possibilities of the runner [53].

## Physiological and cognitive function

Besides aging and a sedentary lifestyle deterioration in physical function, cardiorespiratory fitness and muscle mass have been established to accelerate physiological decline in later decades of life [54]. An individualized training programme can minimize this decline, thus preventing older adults (age 65+ years) from crossing functional thresholds of inability [55]. Vascular function and blood flow regulation form a crucial link between cardiovascular functioning and normal cognitive functions. Moreover, vascular dysfunction appears when blood vessels lose their ability to respond generally due to damage of the endothelial cell layer (caused by advanced glycation end products, inflammation, oxidative stress, etc.) or an increase in the collagen-to-elastin protein ratio within the intimal and medial layers of the vessel wall. Consequently, this disrupts the arterial ability to supply adequate blood flow to the target organs and therefore is at a higher risk of developing into clinical disease [56].

In continuation, dysfunction in one vascular bed is thought to translate to other vascular beds, resulting in systemic vascular dysfunction that will likely alter blood flow to the brain that in time can affect cognitive impairment [44]. Measuring relative changes in oxygenated (O2Hb) and deoxygenated (HHb) hemoglobin in the cortex with the fNIRS method in order to compare sedentary reading control (RC), cognitive exercise (CE), physical exercise (PE) and cognitive + physical exercise (CE + PE) demonstrated that combined variant was superior to single exercise for task-efficient cerebral oxygenation and improved oxygen utilization during cortical activation in older individuals [57].

Moreover, the authors concluded that to maximize cognitive performance, older adults need to undertake more cognitive-demand exercises or more kinds of activity. Unfortunately, the majority of older persons with cognitive dementia were also observed to have vascular dementia with marked leukoariosis (white matter hyperintensities) on CT or MRI [58]. Thus, monitoring vasculature and blood flow regulation to detect dysfunction in the vascular system has high clinical relevance for both cardiovascular disease and cognitive functions.

However, a positive effect on slow cognitive decline is not univocal, and some authors have observed evidence in systematic reviews for the mostly insufficient effectiveness of single-component PA interventions in preventing cognitive decline [59]. Although authors indicate several limitations of their studies in which (i) most analyzed trials were small and did not follow participants long term; (ii) the mode, volume, intensity, and frequency of PA interventions varied widely; (iii) numerous trials enrolled sedentary adults, but inactive status was defined or measured unclearly; (iv) trials used numerous different cognitive tests; and (v) many studies measured cognitive outcomes with various instruments and did not correct for multiple comparisons. Nevertheless, it is necessary to analyze the changing situation of the social structure as the total population ages.

**Figure 1.**
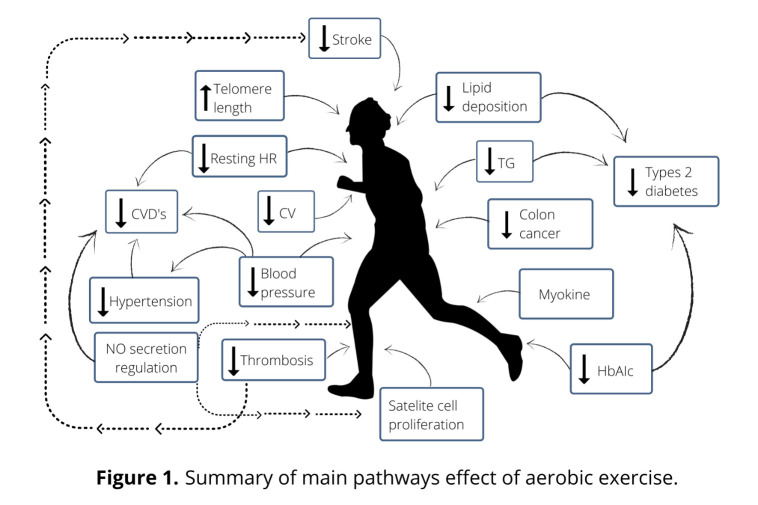
Summary of main pathway effect of aerobic exercise.

## Is exercise a real polypill against cognitive decline?

**Figure 2.**
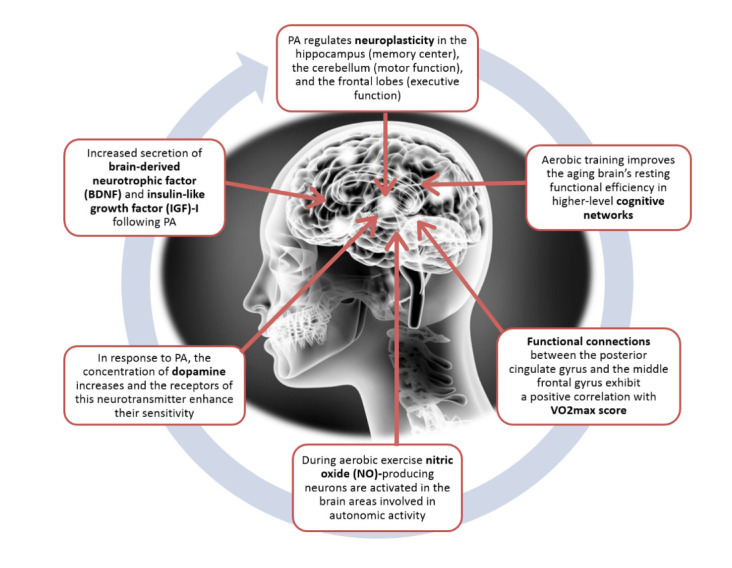
The main pathway effect of aerobic exercise on the central nervous system.

Despite the reservations and skepticism of some authors regarding the positive impact of exercise on healthy aging [59], practice also has a significant effect on the entire architecture of the human body, including physical, psychological and mental dimension (Figure 1). Verghese *et al*. [6] reported on a cohort of 469 subjects over 75 years old in which such leisure activities as reading, playing musical instruments, playing board games and dancing were associated with a reduced risk in dementia. Muscles and nervous tissues are functionally connected, and thus, an exercising person who usually focuses on training muscles also needs to be aware that during exercise, the nervous system is trained as well. The nervous system is characterized by a unique adaptive ability of neuroplasticity, complex molecular and cellular processes that represents the biological basis of the so-called “cerebral reserves”, which can modify itself in response to experience [60]. Exercise and PA may be considered as enhancers as well as environmental factors promoting neuroplasticity (Figure 2). Nervous tissue allows the creation of new connections in the neuronal network and their reorganization, an adaptation ability and biofeedback to such external stimuli as physical exercise (PE) and cognitive exercise.

Numerous studies have demonstrated that, PE stimulates in human adults such structural changes as increased gray matter volume in the frontal and hippocampal regions [61] as well as reduced damage in the gray matter [62]. Thus, physically active people are more likely to maintain cognition in older age in than those who are physically inactive. To date, it remains unknown whether these positive adaptations in the nervous system occur only in healthy adults or also concern such pre-AD stages as mild cognitive impairment (MCI) or severe cognitive deficiency (SCD).

## Stimulation of neural plasticity and cognitive functions through exercise and inclusion of mindfulness and meditation in physical activity classification

There is “no health without mental health” [63]. Dual-task conditions, in comparison to single-task ones, are recognized to improve for exercising older people their postural stability [64], balance and cognition [65]. Makizako et al. [66] coined the term “cognicize” to describe the exercises that are conducted in multitask conditions that include physical and cognitive tasks. However, the idea is to focus on meditation or in broader sense mindfulness as a potentially cognitive task during dual-task exercise. Meditation plays a unique role among numerous non-pharmacologic therapies used to maintain or enhance the cognitive function of the body and mind. Studies on mindfulness, awareness, and meditation are fascinating because they bring knowledge closer to mysterious unknown processes that are still being studied to this day [67].

Although it is not commonly defined or even included in discussions, encyclopedic sources illustrate it as “an intentional and self-regulated focusing of attention that aims to relax and calm the mind and body” [68]. Encyclopedia Britannica focuses on exercise for self-awareness: “private devotion or mental exercise encompassing various techniques of concentration, contemplation, and abstraction, regarded as conducive to heightened self-awareness, spiritual enlightenment, and physical and mental health.” Oxford Learner's Dictionaries associates it with thinking on/upon something and describes it as, “to think deeply, usually in silence, especially for religious reasons or to make your mind calm”. In a more general description, meditation is the practice of using specific techniques such as mindfulness, focusing the mind on a particular object, thought or activity, and so the aim is to train awareness and attention to achieve a mentally and emotionally transparent, stable state [69,70]. All available definitions of meditation focus on the process of calming thoughts and emotions, which is achieved via a comfortable body position and the slow progress of inaction, including thinking. This non-uniform definition is not the only problem since scientific research on this scope shows at least two limitations.

Firstly, meditation studies show that a selection marker or trait is neither known nor given allowing for including/excluding subjects on the protocol. The only criterion of including/excluding individuals is a subjective and non-verifiable opinion; for instance, experienced meditators are defined as meditating ≥30 minutes per day for at least five days per week over the past five years [71]. Secondly, authors do not precisely define the meditation process using such sophisticated research methods as morphometric imaging [67]. Meditation can be classified into three main subclasses depending on body kinesthetics: the sitting position (yoga, zen, Buddhism), the lying position (yoga, vipassana) or in motion (Taijiquan, qigong, whirling dances, circle dances, sustained exercise). The last subclass is crucial in the context of exercising for older people. Meditation can be specified as the disconnection of active consciousness, which may occur in all three mentioned subclasses. In exercise and generally during PA, the third subclass is of particular interest. Recently, Tolahunase et al. [72] found significant improvements in both the cardinal biomarkers of cellular aging [DNA damage marker 8-hydroxy-2′-deoxyguanosine (8-OH2dG), oxidative stress markers reactive oxygen species (ROS), and total antioxidant capacity (TAC), and telomere attrition markers telomere length and telomerase activity] and the metabotrophic biomarkers influencing cellular aging (cortisol, *β*-endorphin, IL-6, BDNF, and sirtuin-1) in apparently healthy population after a 12-week yoga- meditation based lifestyle intervention. Furthermore, such mind-body activities that require attention engagement as yoga, tai chi and meditation have been associated with positive changes in brain structure and function, especially in areas related to awareness, attention, executive functions and memory [73,74,75]. Long-duration exercise not only concerns the state of the body but also changes in the mental state during a bout [76]. Progressive fatigue of the peripheral and then central nervous system is universal regardless of whether it is running, swimming, cycling, prolonged climbing or another mode of exercise, so the exploration of even “millimeter” reserves is relevant. That being said, enthusiasm or motivation can be a crucial aspect of exercising by preventing premature fatigue, though as the consequence/reward due to the refusal to cease training.

Overall, mindfulness meditation during training can be helpful during prolonged efforts. Elements that lead to reaching a state of meditation during exercise are known and are as follows: focusing on inhale-exhale breathing rhythm. Nosal mode and oral mode [77], focusing on cadence (running, biking, hitting) while coordinating bodily movement to the rhythm of the music [78]. This is especially challenging, for men with more difficulties adjusting the cadence of exercise to a musical rhythm.

Rhythm-based music interventions are considered as useful prevention against dementia [79], falls [80], and Parkinson's disease [81]. and moreover, enhance neuroplasticity of the nervous system [82,83]. Rhythmic auditory stimulation (RAS) has been explained in the process of auditory-motor entertainment [84] sensorimotor coupling to temporally structured auditory input, and during recruitment of a striato-thalamocortical-system, involving the thalamus, premotor, the basal ganglia, supplementary motor and dorsolateral prefrontal cortex [85]. Thus, rhythm-based music needs to be recommended for people over 60 years old when exercising.

Visualization techniques from mindfulness meditation may also be recommended for the persons practicing long-duration exercises to shift attention from fatigue and pain to other elements and structures of the body [86]. Thus, we recommend strict and precise psychological phenomenon (processes) focuses attention both on the top of the body (coronal suture) and at the center of the body (plexus Solaris). These points are recommended since they are generally not painfully “destroyed” or “devastated” during a long-lasting bout and may be easily associated with the imagination.

In summary, an exercise model for physically active people over 60 should be individualized according to health status and include mindfulness meditation during movement and visualization during rhythm-based music long-duration exercise in order to prevent against progressive aging and to slow the decline of cognition functions.

## Be balanced

Higher PA can be incorporated with moderate-intensity PA on all days of the week than that recommended by WHO and small hunter-gatherer populations and master athletes can serve as a model group of PA for healthy aging as the next step in rethinking exercise/bout for older adults.

The conventional approach in recent physical activity and fitness clubs is “be balanced” which is understood as psychosomatic harmony, integrity, an optimal mind/psyche level and health-related fitness. Thus, specialists in physical activity and health/fitness coaching must design g individualized exercises and training programs that provide stimulation of motor skills, cognitive functions, interactivity of participants, synergistic impact, external strengthening and evaluation/audit of achieving fitness processes. This issue is of high interest because of the risk of sarcopenia and association of muscle mass with endurance skills since VO_2max_ is mostly in older people dependent on muscle and bone mass as the working muscles stimulate heart adaptation to exercise as well as the vascular system. We propose for older people a moderate-intensity structured training program composed of endurance and resistance exercises, flexibility, falls prevention programs and coordination stimulation in order to obtain an effective cooperation of muscle-nervous tissues.

Cardiorespiratory fitness should be accompanied by searching to be balanced in the context of cognitive functions and mental states.

Furthermore, older people should regularly involve themselves in mind-body activities that require such attention engagement as yoga and meditation [87]. Thus, complex lifestyle intervention program should include challenging physical exercises and mindfulness-based practices that adjusted provide older clients with noticeable changes and progress towards the goal of being balanced.
